# Identification of CAF signature genes and construction of CAF-based risk signature in hepatocellular carcinoma by multi-omics analysis

**DOI:** 10.3389/fimmu.2025.1690174

**Published:** 2025-10-24

**Authors:** Wenchen Qian, Kezhi Wu, Zhu Shen, Bowen Ni, Haojie Zheng, Yawei Liu, Manlan Guo

**Affiliations:** ^1^ Medical Research Center, The Eighth Affiliated Hospital, Southern Medical University (The First People's Hospital of Shunde, Foshan), Guangdong, China; ^2^ Department of Neurosurgery, The Eighth Affiliated Hospital, Southern Medical University (The First People's Hospital of Shunde, Foshan), Guangdong, China; ^3^ Oncology Department, Guilin Hospital of the Second Xiangya Hospital, Central South University, Guilin, Guangxi, China

**Keywords:** hepatocellular carcinoma, cancer-associated fibroblasts, CAF signature genes, CAF-based risk signature, prognosis

## Abstract

**Background:**

Cancer-associated fibroblasts (CAFs) play a critical role in hepatocellular carcinoma (HCC) progression. This study aimed to develop a CAF-based risk signature model for predicting prognosis and identifying potential therapeutic targets.

**Methods:**

Single-cell RNA sequencing (scRNA-seq) and spatial transcriptomic RNA (stRNA) were employed to identify CAF signature genes and their spatial distribution in HCC tissues. Immunohistochemistry (IHC) was used to validate candidate protein expression. A CAFs-based risk signature model was developed using multivariate Cox regression. Functional experiments were performed to evaluate the role of OLFML2B in the effects of CAFs on HepG2 cell proliferation and invasion.

**Results:**

scRNA-seq analysis of dataset GSE242889 found CAFs as pivotal regulators in the HCC microenvironment. Four CAF signature genes (NDUFA4L, OLFML2B, SEMA5B and RASL12) were negatively correlated with HCC patient survival. IHC staining further validated significant upregulation of NDUFA4L, OLFML2B, SEMA5B and RASL12 in HCC tissues. The CAF risk model constructed based on four CAF signature genes demonstrated prognostic predictive value for HCC patients. Moreover, silencing OLFML2B markedly attenuated the CAF-induced proliferation and invasion of HepG2 cells.

**Conclusion:**

This study presents a novel CAF-based risk model that can exhibits accurately predict the prognosis of HCC patients. Furthermore, knockdown of OLFML2B attenuates the CAF-induced HCC progression, suggesting it as a potential therapeutic target.

## 1 Introduction

Hepatocellular carcinoma (HCC) ranks as the fourth most lethal malignancy globally, claiming over 800,000 lives annually due to its high recurrence rates and limited curative options (1). Current therapeutic strategies for HCC mainly include surgery, radiotherapy and chemotherapy. In recent decades, significant progress has been made in understanding the molecular mechanisms promoting HCC progression and in exploring potential therapeutic targets (2–4). However, the heterogeneity of HCC leads to poor patient outcomes. Therefore, identifying novel therapeutic targets remains an urgent clinical need.

The tumor microenvironment (TME) serves as a dynamic orchestrator of HCC progression, where reciprocal interactions between malignant cells and stromal components contribute to tumor growth and therapeutic resistance (5). Cancer-associated fibroblasts (CAFs), predominantly derived from activated hepatic stellate cells, represent the most abundant cell type within the HCC TME (6, 7). CAFs provide a favorable internal environment for the development and malignant progression of HCC by secreting various cytokines, chemokines, and growth factors, either directly or indirectly. Furthermore, CAFs reshape the immune microenvironment by suppressing the activity of immune effector cells and recruiting immunosuppressive cells, thereby enabling cancer cells to evade immune surveillance and leading to poor immunotherapy outcomes (8, 9). Therefore, elucidating the regulatory role of CAFs in HCC is crucial. This not only facilitates a deeper understanding of the mechanisms underlying HCC progression but also holds significant clinical value for assessing patient prognosis and developing targeted therapeutic strategies.

The current study aimed to develop a CAF-based risk signature model for predicting prognosis and immunotherapy response of HCC patients, as well as identifying potential therapeutic targets. Four CAF signature genes (NDUFA4L, OLFML2B, SEMA5B and RASL12) in HCC by utilizing single-cell RNA sequencing (scRNA-seq) and spatial transcriptomics (stRNA) data. Based on these genes, the constructed CAF risk model demonstrated prognostic predictive value for HCC patients. Moreover, functional experiments further revealed OLFML2B as a key regulator of CAF-mediated HCC progression, suggesting it as a promising therapeutic target.

## 2 Materials and methods

### 2.1 Data collection and processing

The scRNA-seq dataset GSE242889 was obtained from the Gene Expression Omnibus (GEO) database (https://www.ncbi.nlm.nih.gov/gds). This dataset included ten samples: five HCC samples and five normal tissue samples (10). To ensure the quality of our analysis, we filtered out single cells expressing fewer than 200 genes or those with any gene expressed in fewer than three cells. Then the percentage of rRNA and mitochondria were calculated using the PercentageFeatureSet function in the Seurat R package. After quality control, a total of 9,029 cells were deemed suitable for further analysis.

In addition, bulk RNA-seq data and clinical metadata for HCC were obtained from The Cancer Genome Atlas (TCGA-LIHC) (https://portal.gdc.cancer.gov/). After excluding samples with missing survival data or outcome status, the dataset included 374 tumor samples and 50 normal samples.

### 2.2 Definition of CAFs

The HCC scRNA-seq data were re-analyzed using the Seurat R package (11) to systematically characterize the CAFs signature. First, cells expressing fewer than 300 or more than 7000 genes were excluded, and the remaining genes were normalized using SCTransformed. The RunHarmony function was employed to remove batch effects for the four samples. Non-linear dimension reduction was performed using the uniform manifold approximation and projection (UMAP) method, and principal components with resolutions of 1.2 and 25 were selected. The single cells were then clustered into different subgroups using the FindNeighbors and FindClusters functions, with parameters set to dim = 25 and resolution = 1.2. UMAP dimensional reduction was further performed using the RunUMAP function. The FindAllMarkers function was used to define marker genes for each CAFs cluster, and comparisons were made between different clusters (minpct = 0.3, logFC = 0.5, and adjusted *P* < 0.05). CAFs were annotated based on two marker genes, including ACTA2 and COL1A2. Additionally, the monocle R software package was used to analyze the developmental trajectory. Finally, Kyoto Encyclopedia of Genes and Genomes (KEGG) enrichment analysis was performed on the marker genes using the clusterProfiler package (12).

### 2.3 CAF-related signature genes identification

DESeq2, edgeR and limma packages (13) were used to identify differentially expressed genes (DEGs) between normal and HCC tissues based on |log_2_(FC)| > 1 and false discovery rate (FDR) < 0.05. The intersection of DEGs and CAF marker genes was subsequently determined. These intersecting genes were further evaluated for prognostic value by generating Kaplan-Meier (KM) survival curves.

### 2.4 stRNA analysis

The stRNA dataset GSE238264 was obtained from GEO database (14). Gene unique molecular identifier counts were normalized and scaled using the Seurat package in the R software. Then spatial alignment was performed to map target gene expression to tissue coordinates, enabling visualization of gene distribution within the tumor microenvironment.

### 2.5 Tissue collection and immunohistochemical assay

Tissue microarray of 30 human hepatocellular carcinoma and paired adjacent normal tissues (HLiv-HCC060PG-01) were purchased from Shanghai Outdo Biotech Company (Shanghai, China). All human tissue studies were approved by the Ethics Committee of Shanghai Outdo Biotech Company (Approval No. SHYJS-CP-1304015).

As previously described (15), paraffin-embedded sections were stained with anti-NDUFA4L2 (Abclonal, Cat# A14288, Wuhan, China), anti-OLFML2B (Abmart, Cat# PA4139, Shanghai, China), anti-SEMA5B (Abmart, Cat# PK92655S), anti-RASL12 (Bioss, Cat# bs-19738R, Beijing, China). Stained sections were examined and imaged using a Leica DM4B microscope system.

### 2.6 Construction and verification of a CAF-based risk signature model

Univariate Cox regression analyses on CAF signature genes were performed using the survival R package. Lasso analyses were applied to remove covariate genes from CAF signature genes. Subsequently, the coefficients were determined by multivariate Cox regression analysis, enabling the construction of a CAF-based risk signature model. Based on this model, HCC patients in the TCGA cohort were stratified into low-risk and high-risk groups using the zero-mean normalization method. KM analysis was used to assess survival differences between the two groups. The prognostic effect of the risk model over a 5-year period was assessed using the receiver operator characteristic curve (ROC).

### 2.7 Nomogram construction

A nomogram integrating the risk signature and clinicopathological features was developed to predict HCC prognosis. Variables with a *P* < 0.05, identified through univariate and multivariate Cox regression analyses, were included in the nomogram. The predictive accuracy of the nomogram was evaluated using calibration curves.

### 2.8 Differentially expressed genes analysis and pathway enrichment analysis

DEGs between low-risk and high-risk HCC groups were identified using the edgeR package, with thresholds of |FC| ≥ 1.5 and adjusted *P* < 0.05. Volcano plots showed changes in expression of all genes, while heatmaps highlighted specific DEGs. Subsequently, enrichment analyses for Gene Ontology (GO) terms and KEGG pathways were performed on the DEGs.

### 2.9 Immune landscape analysis based on the CAFs-based risk signature model

As previously described (16), we utilized the CIBERSORT algorithm to comprehensively assess the correlation between risk profiles and the tumor immune microenvironment (TIME). The estimate R package was employed to calculate the stromal, immune and estimated (stromal + immune) scores, enabling assessment of microenvironmental differences across risk groups. In addition, we estimated the proportion of 22 immune cell subtypes using the TCGA cohort-based CIBERSORT algorithm.

### 2.10 CAFs culture

Human primary liver CAFs were obtained from Meisen Chinese Tissue Culture Collections. CAFs were cultured in DMEM containing 10% FBS. The purity of CAFs was confirmed by immunofluorescence staining. Conditioned media (CM) collected from passages 2–5 of primary CAFs were used for further experiments.

### 2.11 Immunofluorescence

Cells were fixed with 4% formaldehyde and permeabilized with PBS containing 0.1% Triton X-100. Primary antibodies against Vimentin (Proteintech, Cat #60330) or α-SMA (Proteintech, Cat #80008) were incubated overnight at 4 °C. Following incubation with secondary antibody (1 hour, room temperature), nuclei were counterstained with DAPI. Images were captured using a laser confocal microscope.

### 2.12 Cell transfection

CAFs were transiently transfected with 100 nM siRNAs targeting OLFML2B (si-OLFML2B) or control siRNA (NC) using the TransIT-X2 system (Mirus Bio, USA), according to the manufacturer’s instructions. The siRNAs targeting OLFML2B were obtained from GenePharma (Shanghai, China) with the following sequences: NC, UUCUCCGAACGUGUCACGU; si1-OLFML2B, GGACCAACACUCCAAACAATT; si2-OLFML2B, CCACACAGCCACCCAGCAATT; si3-OLFML2B, CCAACUAUUACUACGGCAATT. Transfection efficiency was confirmed by western blotting.

### 2.13 Western blotting

Western blotting was performed as described previously (17). Briefly, protein lysates were separated by SDS-polyacrylamide gels and transferred to PVDF membranes. Primary antibodies included anti-OLFML2B (Abmart, Cat# PA4139) or anti-GAPDH antibody (Abclonal, Cat# AC002). Subsequently, the membranes were incubated with horseradish peroxidase-conjugated secondary antibodies and visualized using enhanced chemiluminescence.

### 2.14 Cell viability assay

HepG2 cells were plated into a 96-well plate at a density of 2 × 10^3^ cells/well and cultured in DMEM medium or corresponding CM. After 24, 48, and 96 hours of incubation, 10 µL of CCK-8 reagent (Dojindo, Japan) diluted in serum-free medium was added to each well. Following 1 hour of incubation, absorbance was measured at 450 nm using a microplate reader.

### 2.15 Cell invasion assay

HepG2 cells pretreated with CM for 48 hours were subjected to invasion assays using 24-well Transwell chambers pre-coated with 6% Matrigel (Corning, USA). A total of 3 × 10^4^ HepG2 cells suspended in 100 µL serum-free DMEM were seeded into the upper chamber, while 600 µL DMEM medium with 5% FBS was added to the lower chamber. After incubation at 37 °C for 24 hours, invaded cells on the lower membrane surface were fixed, stained, and quantified.

### 2.16 Statistical analysis

All statistical analyses were performed using R software (version 4.3.2). The statistical analyses of immunohistochemical staining were performed in GraphPad (version 8.0). The Wilcoxon test was used to compare the two groups, and Spearman or Pearson correlation was used for the correlation matrices. Survival differences in KM curves were evaluated using the log-rank test. Statistical significance was defined as *P* < 0.05; ns, not significant.

## 3 Results

### 3.1 CAFs play a critical role in the HCC tumor microenvironment

**Figure 1:**
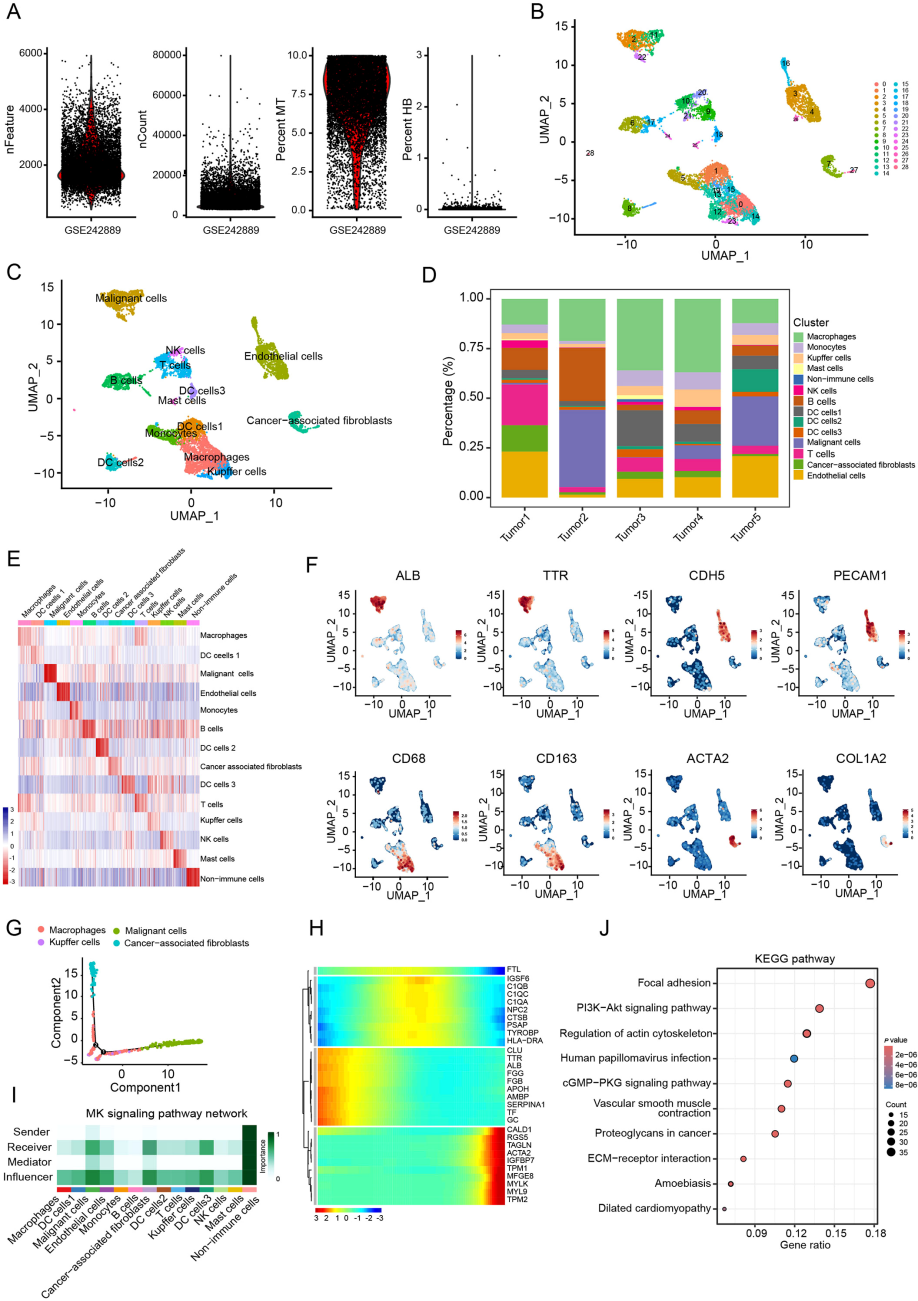
CAFs as a key influencer in the HCC TME. **(A)** nFeature, nCount, mitochondria (MT) and hemoglobin (HB) levels in per cell from the GSE242789 cohort. **(B)** UMAP plots of single cell clusters in HCC tumor. **(C)** UMAP plots of annotated cell types. **(D)** Relative proportions of cell types in HCC tumors. **(E)** Heatmap of gene signatures across cell types (Wilcoxon test). **(F)** UMAP plots of marker genes for malignant cells (ALB and TTR), endothelial cells (CDH5 and PECAM1), macrophages (CD68 and CD163) and CAFs (ACTA2 and COL1A2). **(G)** Trajectory analysis of macrophages, malignant cells, Kupffer cells, and CAFs. **(H)** Heatmap of signature genes along with the pseudotime trajectory. **(I)** Cellular communication analysis in the HCC microenvironment. **(J)** KEGG pathway enrichment analysis of CAF signature genes.

The scRNA-seq expression profile GSE242889 obtained from the GEO datasets was used for analysis. Firstly, the levels of various parameters including feature, count, mitochondria (MT) and hemoglobin (HB) were examined in each cell (**Figure 1A**). After filtering, a total of 9,029 cells were obtained for subsequent analysis. Unsupervised clustering based on gene expression profiles identified 28 distinct cell clusters (**Figure 1B**). These clusters were further annotated into 13 cell subtypes: macrophages, three dendritic cell subsets, cancer cells, endothelial cells, monocytes, B cells, T cells, CAFs, kupffer cells, NK cells and mast cells (**Figure 1C**). Our analysis revealed that CAFs constituted approximately 10% of the total cell population (**Figure 1D**). Subtype annotation accuracy was validated by assessing intra-subset transcriptional homogeneity and marker gene localization. The uniform expression patterns observed within annotated subsets, along with the distinct profiles across subsets, confirmed robust classification (**Figure 1E**). To further confirm the precision of the cell type annotation, we analyzed the distribution of cell-specific markers. This analysis showed that the distribution was correct (**Figure 1F**).

To further investigate intercellular relationships, cell trajectory and cell communication analyses were performed among macrophages, malignant cells, Kupffer cells and CAFs. The cell trajectory analysis revealed that cancer cells occupied the leading position, while malignant cells and CAFs were located at the trailing end of the trajectory (**Figure 1G**). Moreover, CAF signature genes exhibited elevated expression levels in late trajectory phases (**Figure 1H**). Cell-cell communication analysis highlighted CAFs as central interactors within the tumor microenvironment, acting as both primary signal receivers and key influencers in the network (**Figure 1I**). From these analyses, a total of 501 CAF marker genes were identified (**Supplementary Table S1**). KEGG analysis showed that these genes were significantly enriched in pathways such as PI3K-AKT signaling pathway, vascular smooth muscle contraction and ECM-receptor interaction (**Figure 1J**). These findings suggest that CAFs exert pivotal regulatory roles in the HCC microenvironment.

### 3.2 Expression of four CAF signature genes negatively correlates with prognosis in HCC patients

**Figure 2:**
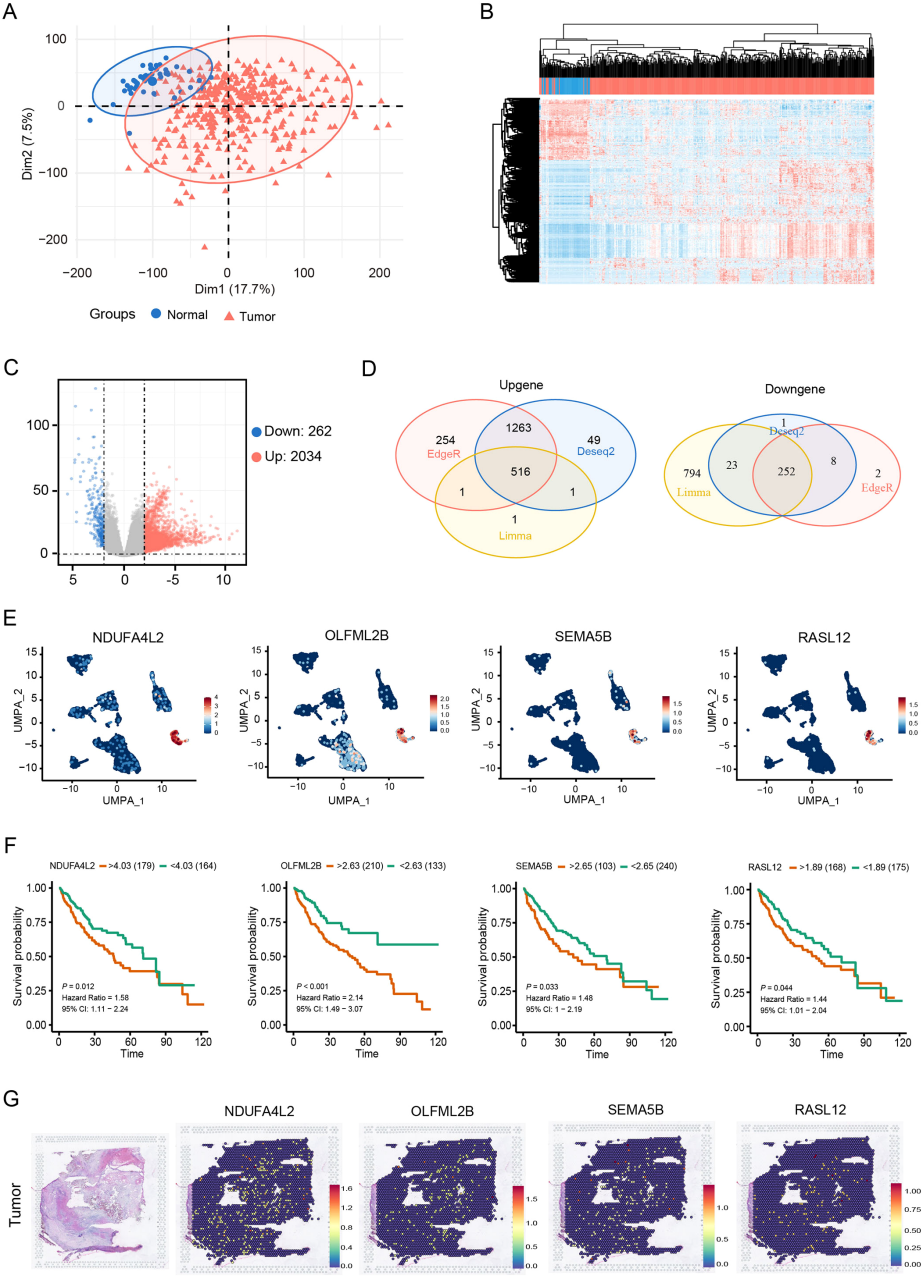
Expression of four CAF signature genes negatively correlates with prognosis in HCC patients. **(A, B)** PCA and clustering heatmap of normal and tumor samples. **(C)** Volcano plot of DEGs between normal and tumor samples. **(D)** Venn diagram of DEGs. **(E)** UMAP plot showing the expression of NDUFA4L2, OLFML2B, SEMA5B and RASL12 in CAFs. **(F)** Survival analysis of NDUFA4L2, OLFML2B, SEMA5B and RASL12 in HCC patients. **(G)** Spatial distribution of the four CAF signature genes in HCC tissues.

To identify CAF signature genes in HCC, DESeq2, edgeR and limma packages were employed to screen for DEGs between normal and HCC tissues. **Figures 2A, B** showed effective separation between normal and HCC tumor samples. Volcano plots further illustrated the global distribution of DEGs (**Figure 2C**). By intersecting these three different R packages, 768 overlapping DEGs were identified (**Figure 2D**). These 768 DEGs were then intersected with the 501 CAF marker genes, yielding 11 candidate CAF signature genes (**Supplementary Table S2**). Prognostic screening of these 11 genes in the TCGA cohort further identified that four CAF signature genes (NDUFA4L2, OLFML2B, SEMA5B and RASL12) were negatively correlated with the survival of HCC patients (**Figures 2E, F**). Genomic profiling of these four genes revealed a low frequency of single-nucleotide variant mutations. Specifically, SEMA5B and OLFML2B exhibited limited co-mutation events, whereas no mutations were detected in NDUFA4L2 or RASL12 (**Supplementary Figure S1**). Moreover, stRNA analyses revealed that the transcript counts in tumor tissues were markedly higher than those in normal tissues (**Supplementary Figures S2A, B**). Further analysis demonstrated that the four CAF signature genes exhibited a uniform distribution and elevated expression in HCC tissues compared with normal tissues (**Figures 2G**, **S2C**). Together, these findings suggest that the four CAF signature genes are actively involved in the progression of HCC.

### 3.3 CAFs signature genes are highly expressed in HCC tissues

**Figure 3:**
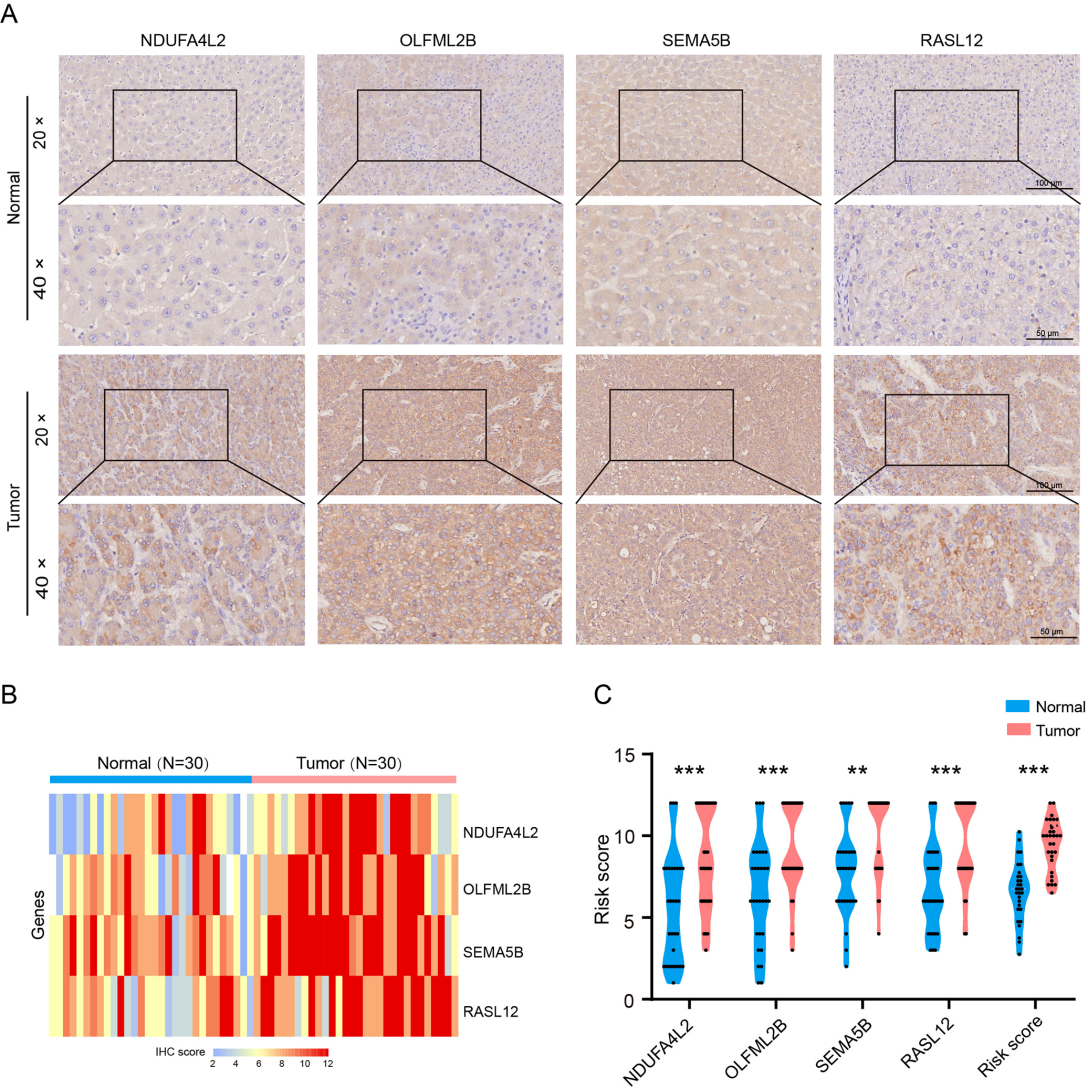
Expression levels of CAF signature genes in paired adjacent normal and HCC tissues. **(A)** NDUFA4L2, OLFML2B, SEMA5B and RASL12 staining. **(B, C)** Quantification of NDUFA4L2, OLFML2B, SEMA5B and RASL12 expression (n = 30). Data are presented as mean ± S.D., **P < 0.01, ***P < 0.001, compared to the normal group.

Immunohistochemical staining was performed to validate the expression of CAFs signature genes in human HCC tissues and paired adjacent normal tissues. The results showed that the levels of NDUFA4L2, SEMA5B, OLFML2B and RASL12 were significantly increased in HCC tissues compared to the normal group (**Figures 3A–C**). These data indicate that CAF signature genes may serve as potential therapeutic targets for HCC.

### 3.4 CAFs-based risk signature model exhibits significant prognostic predictive value for HCC patients

**Figure 4:**
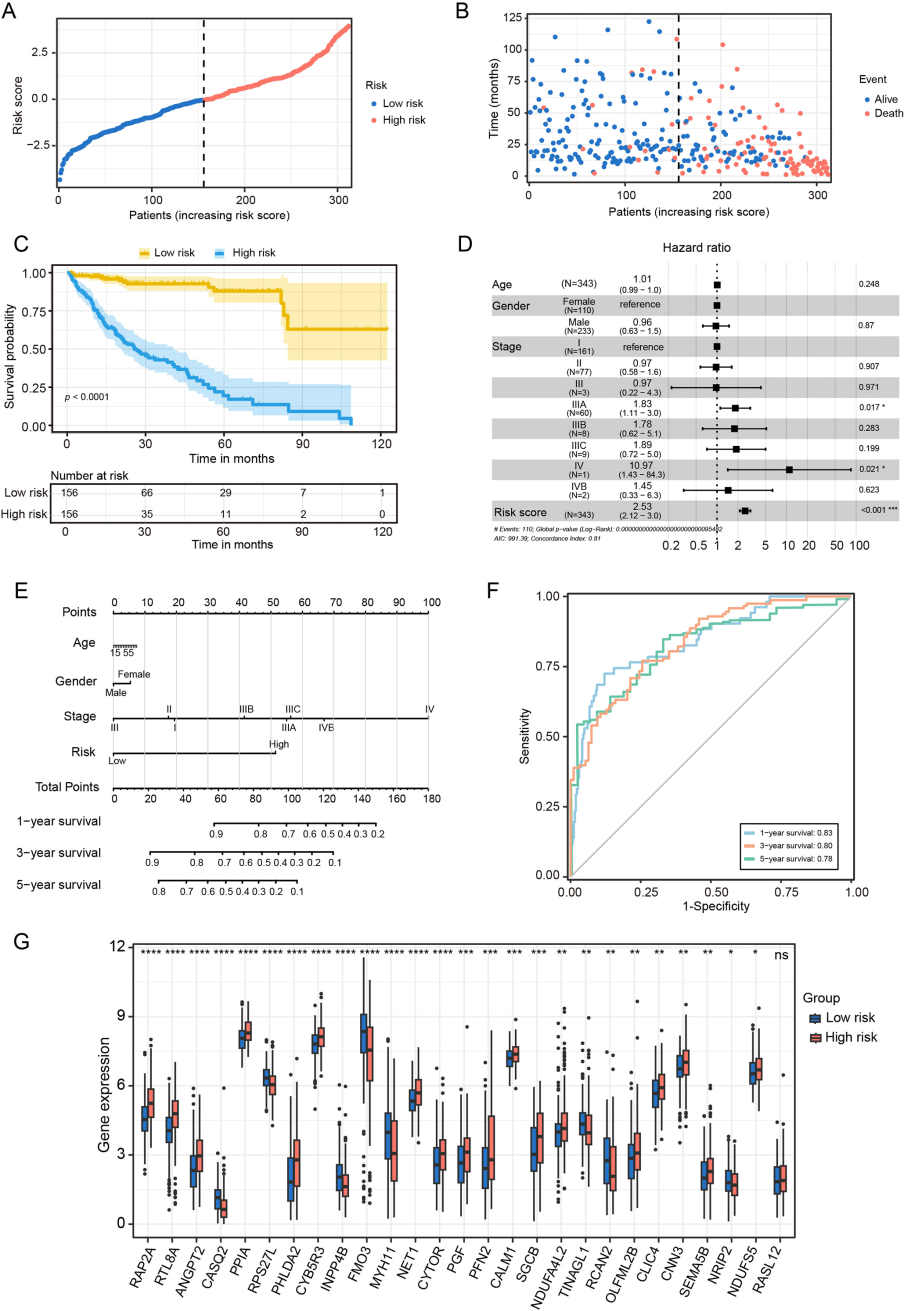
Prognostic value of CAF-based risk signature model in HCC. **(A)** Classification of TCGA HCC patients into low- and high- risk groups based on the risk model. **(B)** Survival status of HCC patients in between low- and high-risk groups. **(C)** KM curves of the risk signature. **(D)** Multivariate COX regression analysis for age, gender, stage and risk score. **(E)** The nomogram for predicting 1-, 3-, and 5-years overall survival. **(F)** ROC curves of the nomogram. **(G)** Statistical analysis of differences in CAFs signature genes between low- and high-risk groups. n.s. no significant difference, *P < 0.05, **P < 0.01, ***P < 0.001 compared to the low-risk group.

To evaluate the prognostic utility of the CAF risk signature model, HCC patients in the TCGA cohort were stratified into low- and high-risk groups (**Figure 4A**). KM survival analysis revealed that patients in the low-risk group exhibited both longer survival times and higher survival probabilities compared with those in the high-risk group (**Figures 4B, C**). Multivariate Cox regression analysis was performed on variables such as age, gender, tumor stage, and risk score. This confirmed the risk score serves as an independent prognostic predictor in the TCGA cohort (**Figure 4D**).

To further enhance clinical applicability, we constructed a nomogram that integrated risk scores with clinicopathological variables to predict 1-, 3-, and 5-year overall survival in HCC patients (**Figure 4E**). Calibration plots demonstrated strong concordance between predicted and observed survival outcomes, indicating robust predictive performance of the model (**Figure 4F**). Furthermore, gene expression profiling revealed that RAP2A, NDUFA4L2, OLFML2B and SEMA5B were markedly upregulated in the high-risk group, whereas CASQ2, FMO3 and MYH11 were overexpressed in the low-risk group (**Figure 4G**). These findings underscore the clinical significance of the CAFs risk signature model in terms of prognostic stratification for HCC patients.

### 3.5 Pathway enrichment analysis of DEGs in the CAF-based risk signature model

**Figure 5:**
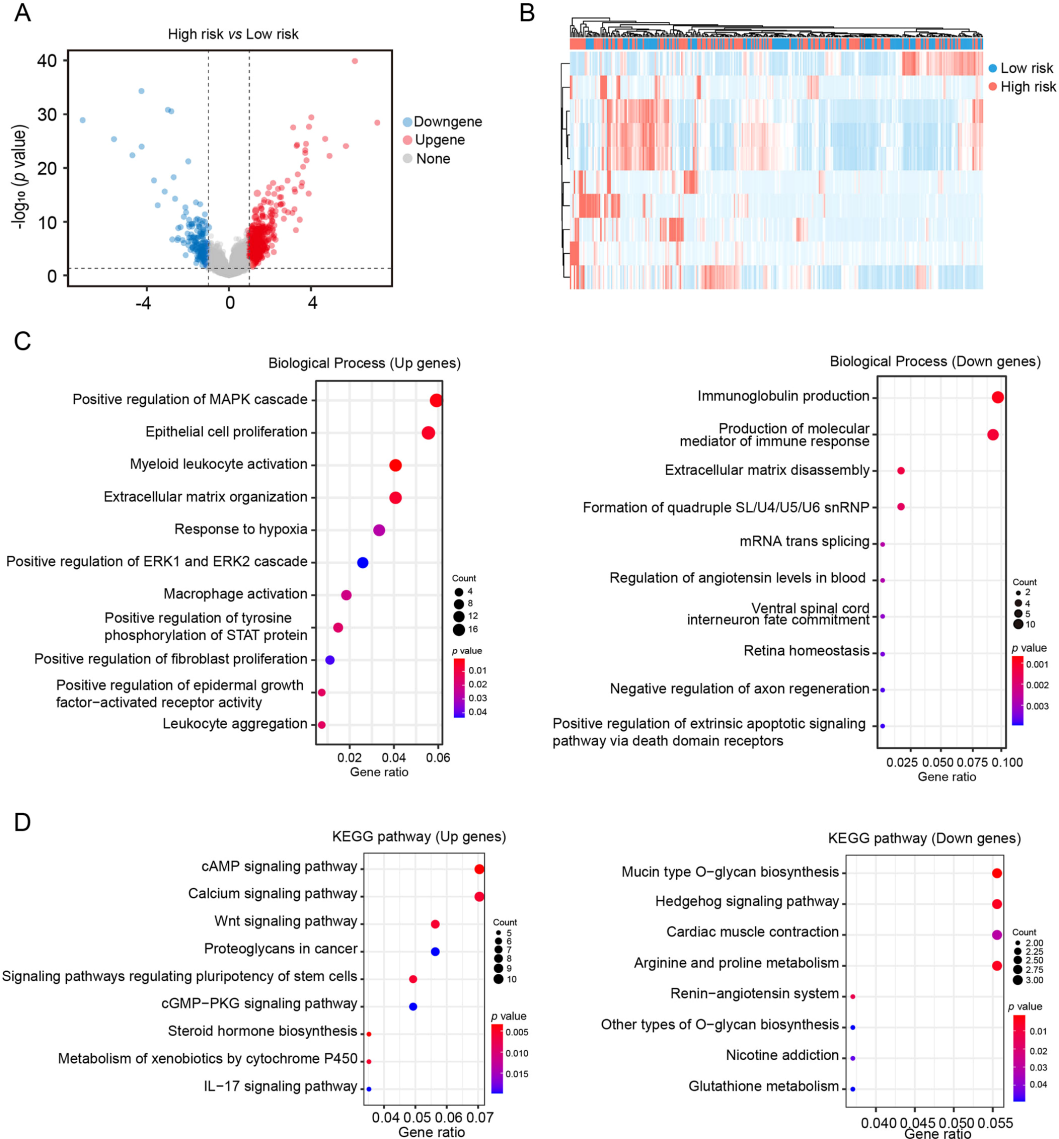
Pathway enrichment analysis of DEGs in the CAF-based risk signature model **(A)** Volcano plot of gene expression differences between low- and high- risk groups in the TCGA HCC cohort. **(B)** Heatmap of gene expression differences between low- and high- risk groups in the TCGA HCC cohort. **(C, D)** GO-BP and KEGG enrichment analysis of DEGs.

The TCGA cohorts was utilized to identify DEGs between low-risk and high-risk groups. As **Figures 5A, B** show, A total of 623 DEGs were identified, including 399 upregulated and 224 downregulated genes in the high-risk group. GO enrichment analysis indicated that upregulated genes were primarily involved in inflammation, hypoxia and fibrosis processes, including myeloid leukocyte organization, macrophage activation, extracellular matrix organization and response to hypoxia. Conversely, downregulated genes were enriched in pathways related to immunoglobulin production and immune mediator synthesis (**Figure 5C**). KEGG pathway enrichment analysis revealed that the cAMP signaling pathway, calcium signaling pathway, and Wnt signaling pathway were significantly upregulated in the high-risk group, whereas mucin type O-glycan biosynthesis and Hedgehog signaling pathway were significantly downregulated (**Figure 5D**). The results suggest that the CAF-based risk signature prediction model provides valuable insights for exploring the molecular mechanisms underlying HCC progression.

### 3.6 Association between CAF-related risk and the immune landscape in HCC patients

**Figure 6:**
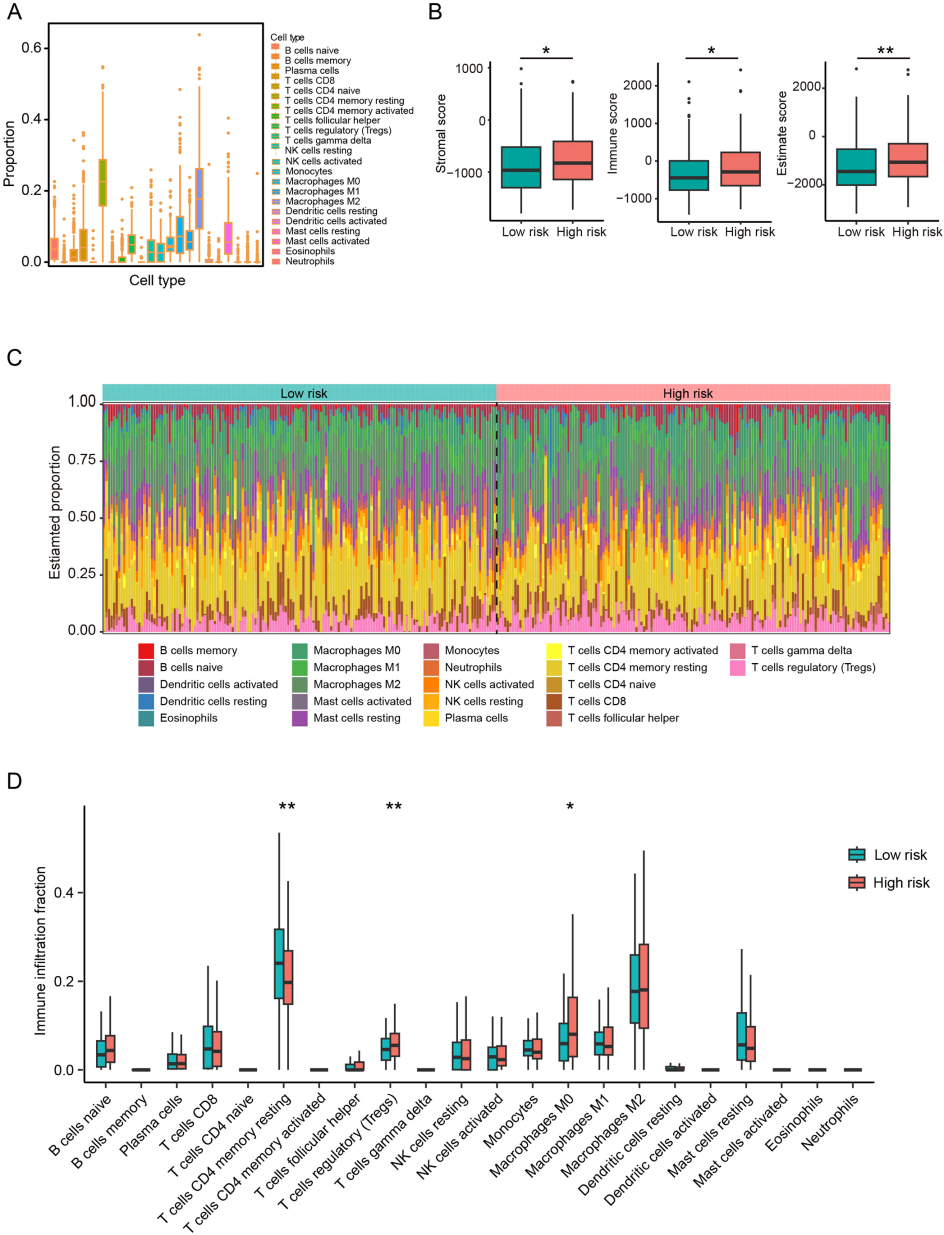
CAFs-based risk signature model predicts immunotherapy response in HCC patients. **(A)** Comparison of the proportions of 22 immune cell types in HCC tissues from the TCGA cohort. **(B)** Stromal, immune and estimate scores between low- and high-risk groups. **(C, D)** Proportions and differences of 22 immune cell types between low- and high-risk groups. *P<0.05, **P<0.01 compared to the low-risk group.

To further evaluate the clinical utility of the CAF-based risk signature model, we assessed its predictive value for immunotherapy responsiveness. ESTIMATE analysis revealed significantly elevated stromal, immune, and combined (stromal + immune) scores in the high-risk group of the TCGA cohort (**Figures 6A, B**). Furthermore, TIMER analysis indicated an increase in various cell types in the high-risk HCC group, including regulatory T cells regulatory and M0 macrophages, compared to the low-risk group, whereas CD4 resting memory T cells were significantly more abundant in the low-risk group (**Figures 6C, D**). In summary, these findings suggest that high-risk patients are characterized by an immunosuppressive TME, which may influence their responsiveness to immunotherapy.

### 3.7 OLFML2B knockdown in CAFs attenuates its tumor-promoting effects on HepG2 cell proliferation and invasion

**Figure 7:**
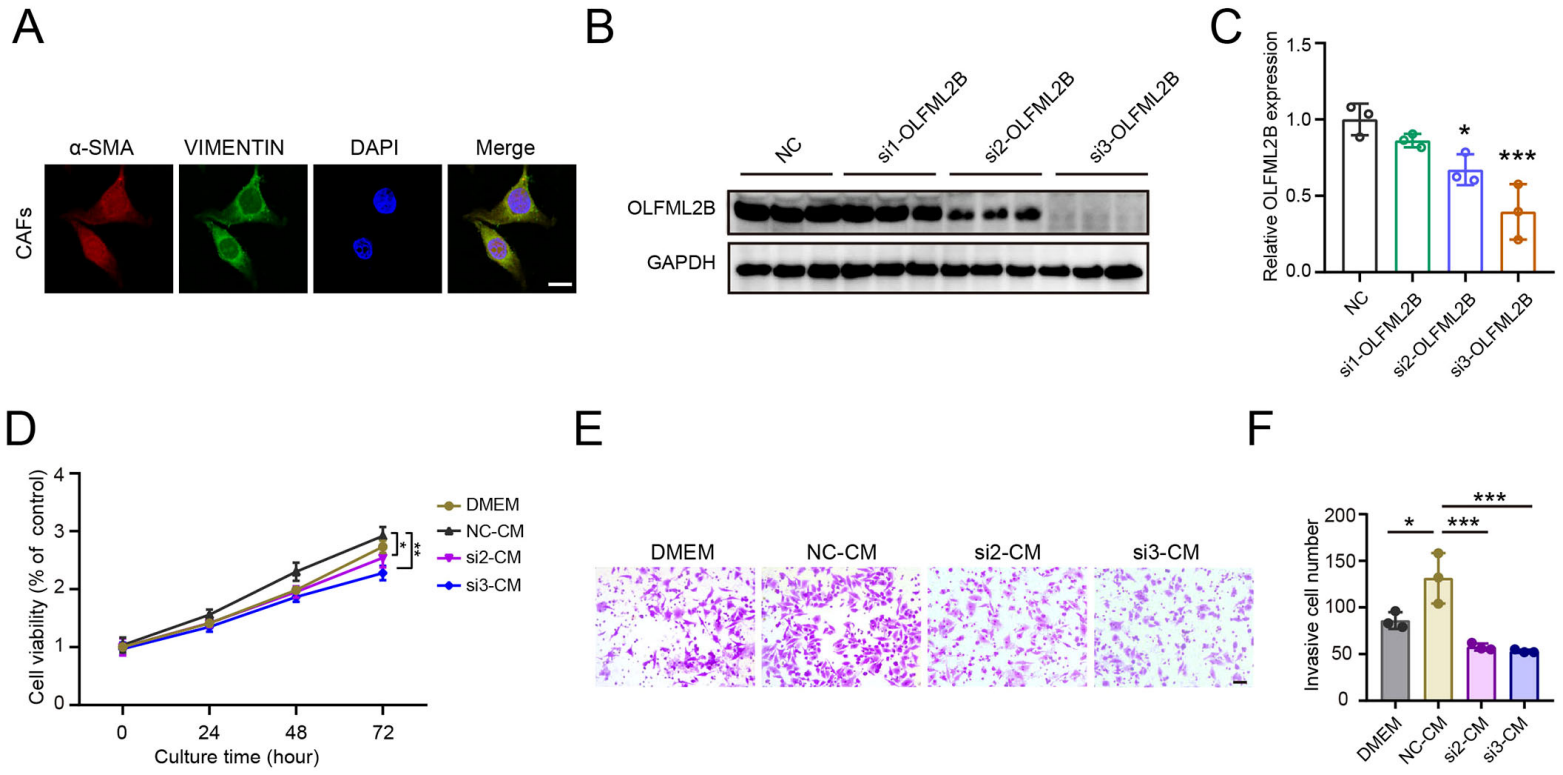
Knockdown of OLFML2B attenuates CAF-induced proliferation and invasion of HepG2 cells. **(A)** Immunofluorescence staining of α-SMA, VIMENTIN and DAPI in CAFs isolated from HCC mice. Scale bars = 100 μm. **(B)** Western blot analysis of OLFML2B expression in CAFs transfected with OLFML2B-targeting siRNAs. **(C)** Quantification of OLFML2B expression (n =3). **(D)** CCK-8 assay showing the proliferation of HepG2 cells cultured with CM from control or OLFML2B-silenced CAFs (n =3). **(E, F)** Transwell assay demonstrating the invasion of HepG2 cells cultured with CM from control or OLFML2B-silenced CAFs. Scale bars = 20 μm. (n =3). Data are presented as mean ± S.D., *P < 0.05, ***P < 0.001 compared to the control group.

Given the strong association between OLFML2B overexpression in CAFs and poor prognosis in HCC, we next evaluated its functional role using siRNA-mediated knockdown. Among the three siRNAs used, siRNA2 and siRNA3 significantly reduced OLFML2B protein levels in primary CAFs (**Figure 7A**). CM collected from control CAFs and OLFML2B-silenced CAFs were used to treat HepG2 cells. CCK-8 assays revealed that OLFML2B-silenced CAFs significantly suppressed HepG2 cell proliferation compared to control CM (**Figure 7B**). Similarly, Transwell invasion assays demonstrated that control CAF-CM promoted HepG2 invasiveness, whereas OLFML2B knockdown abrogated this effect (**Figures 7C, D**). These results demonstrate that OLFML2B is a key mediator of CAF-driven tumor progression, underscoring its potential as a therapeutic target for disrupting CAF–cancer cell crosstalk in HCC.

## 4 Discussion

HCC ranks among the most prevalent malignancies globally worldwide and is characterized by high morbidity and mortality rates. Despite significant therapeutic advances in recent years, the prognosis of HCC patients remains poor, highlighting the need for novel therapeutic strategies (18, 19). Increasing evidence indicates that the TME, particularly CAFs, plays a pivotal role in hepatocarcinogenesis and disease progression (20, 21). However, the lack of validated CAF signature genes has limited the development of effective CAF-targeted interventions. Therefore, it is of critical importance to construct a new CAF risk model for predicting the prognosis of HCC patients and exploring potential therapeutic targets. In this study, we employed multi-omics analysis to identify four CAF signature genes and established a CAF-based risk model that showed significant prognostic value for HCC patients. Furthermore, functional validation confirmed OLFML2B as a key regulator of CAFs-mediated HCC progression, indicating its potential as a therapeutic target.

CAFs, as the most abundant component in HCC, play a crucial role in tumor progression and patient prognosis by regulating intercellular communication and remodeling the extracellular matrix (ECM). Recently, integrated multi-omics analyses have been employed to unravel the heterogeneity of CAF clusters in HCC and to construct various CAF-based prognostic models for predicting patient outcomes. Previous studies have constructed CAF-related genes based on RNA-seq data and Microarray data, utilizing algorithms xCell and MCPcounter, as well as WGCNA, and subsequently validated their potential in prognostic assessment of HCC (22, 23). Yu et al. identified six candidate genes associated with CAF clusters using scRNA-seq and bulk RNA-seq data and constructed a CAF-related risk model for prognostic prediction in HCC (24). These studies have provided valuable insights into the role of CAFs in HCC. However, the identification of CAF-related genes was primarily based on statistical associations, lacking experimental validation. In this study, we integrated scRNA-seq, stRNA and bulk RNA-seq to systematically define marker genes. Through rigorous DEG intersection analysis and survival- association screening, we identified four key CAF-signature genes (NDUFA4L2, OLFML2B, SEMA5B, and RASL12). Furthermore, we validated their expression at the protein level using IHC. Previous studies have reported that high expression of these genes is associated with poor prognosis in multiple cancers, including glioma, gastric cancer and colorectal cancer, and mediates resistance to HER2-positive breast cancer (25–29). In addition, knockdown of NDUFA4L2 in HCC cells has been shown to suppress tumor growth and metastasis (30), which is consistent with our findings of elevated NDUFA4L2, OLFML2B, SEMA5B, and RASL12 expression in HCC tissues and their significant negative correlation with patient survival. In summary, our findings highlight the value of these CAF-signature genes as potential therapeutic targets in HCC. The multi-dimensional data integration and experimental validation provide evidence to support the practical application of a CAF-based risk model for predicting HCC prognosis.

Our GO and KEGG enrichment analysis revealed pathways such as ECM–receptor interaction, response to hypoxia and Wnt signaling, all of which are functionally relevant to HCC. The ECM–receptor interaction pathway has been shown to promote ECM remodeling and enhance invasive capacity of HCC cells (31). Hypoxia-related responses are also critically involved in HCC, as hypoxic stress induces HIF-1α–dependent transcriptional programs that facilitate angiogenesis, metabolic reprogramming and immune evasion, ultimately contributing to poor prognosis (32). In addition, dysregulated Wnt/β-catenin signaling has been linked to stemness, tumor initiation, and therapy resistance in HCC (33). Taken together, these findings indicate that the enriched pathways are mechanistically linked to HCC progression, thereby reinforcing the biological relevance of our CAF-based signature.

CAFs closely interact with immune cells in the HCC stroma to stimulate their pro-tumorigenic capacity. Studies have found that CAFs promote immune tolerance in HCC by conferring tolerogenic characteristics on Dendritic Cells, promoting T cell anergy, and inducing regulatory T cells (Tregs) differentiation (34, 35). In this study, the high-risk group exhibited higher levels of immune cell infiltration and a more significant infiltration of immunosuppressive Tregs, aligning with previous findings by Dong et al (36). These findings may be beneficial for the development of personalized immunotherapy strategies.

OLFML2B is an extracellular matrix protein containing an olfactomedin-like domain, which plays a critical role in intercellular adhesion and tumorigenesis. Studies have shown that OLFML2B is upregulated in multiple cancers, and its high expression is closely associated with poor prognosis, tumor stage, TME remodeling, and immune cell infiltration (37–39). In recent years, OLFML2B has been found play a significant role in the progression of HCC. It holds promise as a diagnostic biomarker, particularly showing potential in predicting incidence of HCC during the cirrhotic stage (40). Moreover, the latest research has revealed that OLFML2B is upregulated in senescent CAFs and identified as one of the key prognostic genes associated with senescent CAFs. A CAF senescence score model, constructed based on OLFML2B and nine other genes, can accurately predict the prognosis of HCC patients (41). Although previous studies have identified the association between OLFML2B and the malignant progression of HCC, its underlying oncogenic mechanisms remain unclear. In the present study, we demonstrate that knockdown of OLFML2B in CAFs attenuates its tumor-promoting effects on HepG2 cell proliferation and invasion, suggesting that OLFML2B is a potential therapeutic target in HCC.

In summary, we used open scRNA-seq and stRNA data to identify four CAF signature genes (NDUFA4L2, OLFML2B, SEMA5B and RASL12) significantly negatively associated with HCC. The CAF-based risk signature model predicts survival and reflects the immune landscape of the TME. Furthermore, functional validation revealed that OLFML2B is a key regulator of CAF-mediated HCC progression. Together, our findings provide novel insights into CAF-driven mechanisms and potential therapeutic targets in HCC.

## Data Availability

The original contributions presented in the study are included in the article/**Supplementary Material**. Further inquiries can be directed to the corresponding authors.
