# Supplementary material for: Identification of CAF signature genes and construction of CAF-based risk signature in hepatocellular carcinoma by multi-omics analysis
Source: Front Immunol. 2025 Oct 24;16:1690174. doi: 10.3389/fimmu.2025.1690174 (PMC12591973; doi:10.3389/fimmu.2025.1690174)
Supplement: Supplementary file 1 [file DataSheet1.docx]

**Supporting Information for**

**ORIGINAL ARTICLE**

**Identification of CAF signature genes and construction of CAF-based risk signature in hepatocellular carcinoma by multi-omics analysis**

**
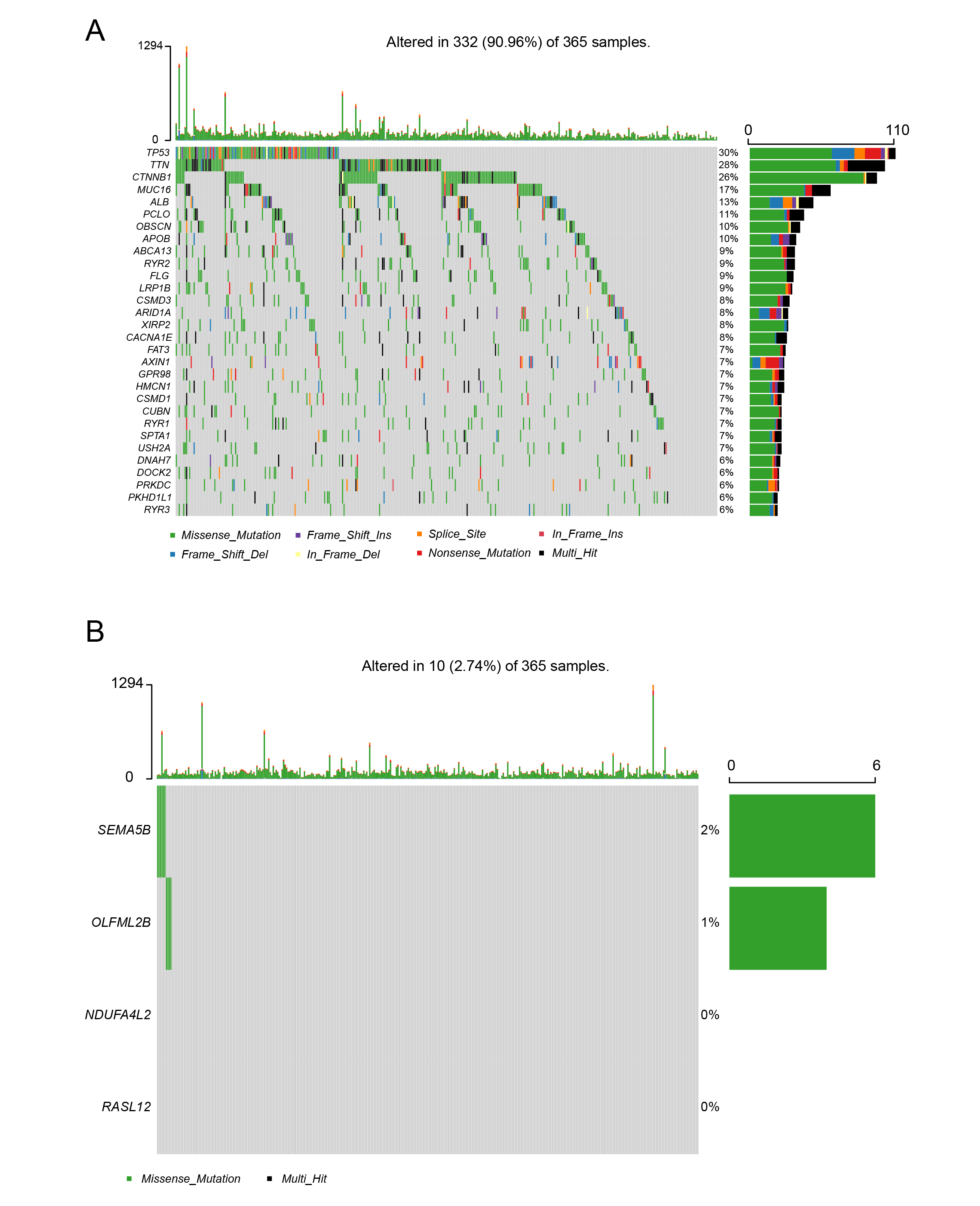
**

**Figure S1** Mutation analysis of CAFs signature genes. (A) The mutation landscape based on the TCGA cohort. (B) Waterfall diagram showing single-nucleotide variants mutations of four CAFs signature genes.


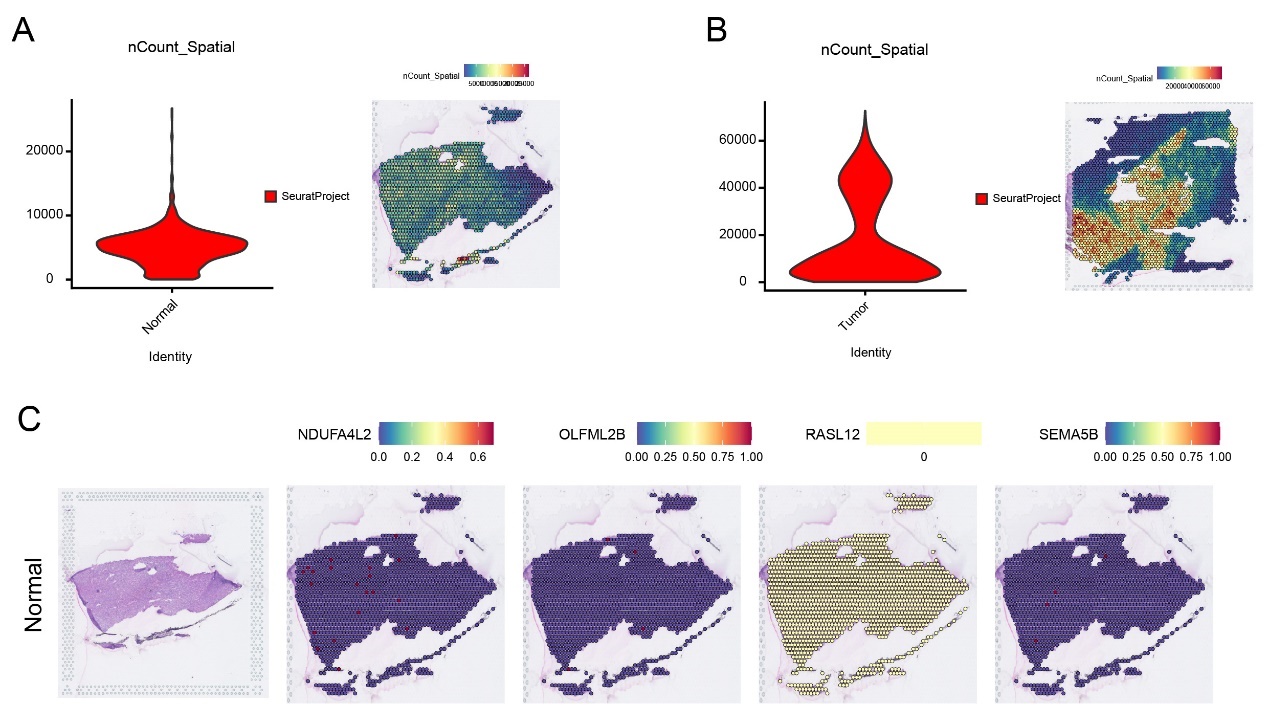


Figure S2 Spatial transcriptome examination in nomal and HCC tissues. (A, B) Evaluation of nCount_spatial for quality and gene quantity. (C) Spatial distribution of the four CAFs signature genes in nomal tissues.
